# Black Phosphorus/Carbon Nanoframes for Efficient Flexible All-Solid-State Supercapacitor

**DOI:** 10.3390/nano12193311

**Published:** 2022-09-23

**Authors:** Zunbin Duan, Danni Liu, Zhaoer Ye, Caixia Sun, Zikun Wang, Kezhen Chen, Yang Li, Hao Huang, Xiaoliang Zeng, Jiahong Wang, Rong Sun, Xue-Feng Yu

**Affiliations:** 1 Shenzhen Engineering Center for the Fabrication of Two-Dimensional Atomic Crystals, Shenzhen Institute of Advanced Technology, Chinese Academy of Sciences, Shenzhen 518055, China; 2 Nano Science and Technology Institute, University of Science and Technology of China, Suzhou 215125, China; 3 Department of Hematology, Zhanjiang Central Hospital, Guangdong Medical University, Zhanjiang 524045, China; 4 University of Chinese Academy of Sciences, Beijing 100049, China; 5 Shenzhen Institute of Advanced Electronic Materials, Shenzhen Institute of Advanced Technology, Chinese Academy of Sciences, Shenzhen 518055, China

**Keywords:** black phosphorus, supercapacitor, carbon nanoparticle, flexible all-solid-state device

## Abstract

A flexible all-solid-state supercapacitor with fast charging speed and high power density is a promising high-performance energy storage and sensor device in photovoltaic systems. Two-dimensional black phosphorus (BP) is a prospective electrode nanomaterial, but it struggles to fully exert its properties limited by its self-stacking. Herein, by embedding carbon nanoparticles into the interlayer of BP microplates, the designed BP/carbon nanoframe (BP/C NF) forms a certain nano-gap on the substrate for promoting the orderly transport of charges. The corresponding supercapacitor BP/C SC has a capacity of 372 F g^−1^, which is higher than that constructed from BP microplates (32.6 F g^−1^). Moreover, the BP/C SC exhibits good stability with a ca. 90% of capacitance retentions after 10,000 repeated bending and long-term cycles. Thus, the proposed strategy of using BP/carbon nanoframes is feasible to develop exceptional flexible energy devices, and it can guide the design of relevant two-dimensional nanocomposites.

## 1. Introduction

With the development of wearable electronics for solar and other clean energy, supercapacitors (SCs) have attracted attention in energy storage and conversion due to their satisfactory light weight, high safety, and easy integration [1,2,3,4]. SCs exhibit unique properties such as fast and stable output, high power density, low cost, and long lifetime, which make them appropriate as energy storage units in solar photovoltaic power generation systems [5,6,7]. Moreover, SCs can be constructed on flexible substrates with suitable solid-state electrolytes for wearable electronics as flexible storage units or pressure-sensing components [8,9,10]. Thus, flexible SCs can potentially be used in solar energy storage systems to convert unstable solar energy quickly and efficiently. In a typical sandwich-structured flexible SC, the electrode material is a crucial component that affects the device’s properties [11,12]. At present, numerous nanomaterials with a large surface area and high conductivity are used as high-performance electrode materials, including carbon nanotubes [13,14,15,16], metal oxides [14,17], metal sulfides [18,19,20], and conductive polymers [21,22]. Among them, two-dimensional (2D) nanomaterials [23,24,25,26,27] with a thickness of only a few atoms possess the characteristics of abundant surface sites, excellent mechanical properties, and high adhesion to substrates, which are increasingly being developed. Therefore, designing 2D nanomaterial electrodes with extraordinary properties could greatly facilitate the progress of flexible SCs.

Black phosphorus (BP), a rising 2D material composed of folded six-membered rings of phosphorus atoms, possesses the gratifying properties of high electrical conductivity and a tunable electronic structure for energy storage, solar thin film, and optoelectronic devices [28,29,30,31,32,33]. In particular, an interlayer spacing as high as 5.3 Å is favorable for ion intercalation and diffusion [34,35]. It is particularly important to prepare nano-scale BP suitable for supercapacitive applications by appropriate means [36]. Hao et al. [37] fabricated a supercapacitor using ultrasonically exfoliated BP nanosheets with a capacity of 45.8 F g^−1^. However, due to the van der Waals forces between the layers, the BP nanosheets are prone to self-stacking, and cannot fully exert their potential electrochemical capacity [25,38]. Our group [26] synthesized ultrathin (<4 nm) BP sponges and constructed a SC with a capacitance of 80 F g^−1^. The capacitive properties of BP sponges are greater than those of BP nanosheets. BP is affected by the easily adsorbed solvent molecules during exfoliation, resulting in a decrease in electrical conductivity [39]. Using inert atmospheres and oxygen-free solvents are the general strategy for protecting BP from degradation [38,40]. On the premise of not destroying the mechanical properties of flexible capacitors, one of the effective ways to enhance the BP’s properties is to increase the effective area of the electrode, promote the orderly transport of charges, and enhance the electrical conductivity.

Herein, we design a rational 2D BP/0D carbon nanoframe (BP/C NF) to effectively improve the supercapacitor performance of black phosphorus. The ultrathin BP microplates obtained by electrochemical intercalation are uniformly mixed with carbon nanoparticles (C NPs) and sprayed on the conductive substrate to form flexible electrodes. The prepared large-size BP improves the contact area between the layers, and the embedded C NPs avoid the self-stacking of almost unoxidized BP to a large extent, as well as promoting the infiltration and diffusion of the electrolyte. Compared with the bare black phosphorus capacitor (BP SC), a flexible all-solid-state supercapacitor (BP/C SC) based on BP/C NF exhibits better electrochemical properties and a gratifying capacitance retention of 93.2% after 10,000 flat-bend tests. It is of great significance to design and fabricate black phosphorus/carbon nanocomposites for high-performance flexible devices and other applications.

## 2. Experimental Section

### 2.1. Materials and Instruments

BP crystals were purchased from Mophos (*www.Mophos.cn*; accessed on 22 September 2022). *N*,*N*-Dimethylformamide (DMF; 99.9%, GC grade), tetrabutylammonium bromide (99.0%, AR grade), ethanol (99.8%, electronic grade), polyvinyl alcohol (PVA 105; 99.9%, GC grade), phosphoric acid (H_3_PO_4_; 99.9%, GC grade) and polydimethylsiloxane (PDMS) were purchased from Aladdin and used without further purification. C NPs (ECP600JD; size, ca. 30 nm shown in Figure S1a) were purchased from Taobao Electrochemical Experimental Instrument Consumables Store. The physicochemical properties are characterized using scanning electron microscopy (SEM), transmission electron microscopy (TEM), atomic force microscopy (AFM), optical microscopy, powder X-ray diffraction (XRD), Raman scattering microscopy, and X-ray photoelectron spectroscopy (XPS), and the corresponding test parameters are shown in the Supporting Information.

### 2.2. Preparation of Flexible All-Solid-State Supercapacitors

BP microplate–ethanol suspension [29]: The BP microplates required for the experiment were prepared in an electrolyte containing 125 mM tetrabutylammonium bromide in DMF with BP crystals as the cathode and platinum sheets as the anode. The voltage between the two electrodes was controlled at 30 V. The swelling product was collected after stripping for 5 min and washed three times with DMF. The products in the DMF suspension of 800–13,000 rpm centrifugation were collected, washed with ethanol 5 times, and finally placed into a BP microplate–ethanol suspension with a concentration of 500 μg mL^−1^.

Solid electrolyte [37]: 3 g of PVA 105 was added to 30 mL of deionized water (>18.25 MΩ) at 85 °C and completely dissolved after being stirred. Then, 10 mL of phosphoric acid was gradually added and stirred until a homogeneous solution was formed. Finally, the electrolyte was cooled to RT for use.

Flexible supercapacitors with black phosphorus/carbon nanoframes: In total, 0.5 mL of ethanol suspension containing 500 μg mL^−1^ BP microplates was mixed with 0.5 mL of C NPs–ethanol suspension (500 μg mL^−1^) and sonicated uniformly, which was used as the active material of the electrode. The ITO-PET substrate (3 cm × 1 cm; thickness, 125 nm for PET film, 185 nm for ITO film; South China Xiangcheng Technology Co., Ltd., Yiyang, China) was soaked in acetone and ethanol for 5 min, and dried with argon gas as the flexible conductive substrate. A piece of ITO-PET substrate was placed on a heating table at 65 °C, and then 0.5 mg of the active material was uniformly sprayed with an area of 1 cm^2^ to obtain the capacitive electrode BP/C NF. To quickly complete the spraying to ensure the stability of the BP microplates, an airbrush with a diameter of 2 μm was used with a HF-600 air pump sprayer. 20 μL of solid electrolyte was flat-coated onto the prepared BP/C NF and baked until dry. One of the two electrodes was coated with 2.5 μL of solid electrolyte for pre-infiltration. The two electrodes were stacked together, dried, and embedded with PDMS to prepare the flexible all-solid-state supercapacitor with an active component loading of 1 mg, which has been labeled as BP/C SC. The compared supercapacitor BP SC was prepared based on the aforementioned steps without the addition of C NPs.

## 3. Results and Discussions

### 3.1. Material Synthesis and Characterization

**Figure 1:**
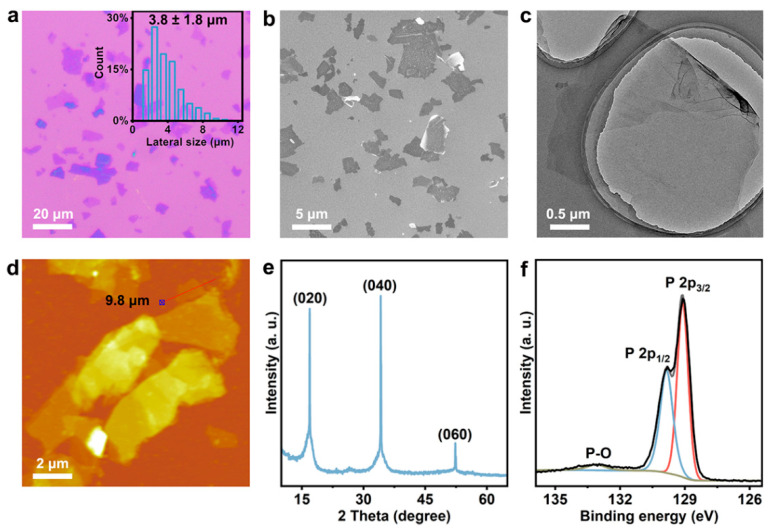
Characterization of BP microplates. (**a**) Photomicrograph of the BP microplates with an insert of lateral diameter statistics. (**b**) SEM image of the BP microplates. (**c**) TEM image of the BP microplate. (**d**) AFM image of the BP microplates with a height profile. (**e**) XRD pattern of the BP microplates containing the characteristic crystal planes of BP. (**f**) P2p XPS spectrum of the BP microplate.

The BP microplates were obtained by electrochemical intercalation and have been characterized by various analytical methods. As shown in Figure 1a, the BP microplates exhibite a relatively consistent color under the optical microscope, mainly light blue, indicating their moderately uniform thickness. The corresponding statistical analysis in the insert of Figure 1a shows an average lateral diameter of 3.8 ± 1.8 μm for the BP microplates. The morphology is further studied by SEM, TEM, and AFM. The microplates exhibite a smooth surface, and the monolayer area is about 10 μm^2^ (Figure 1b,c). The thickness of the BP microplates is 9.8 nm, as evidenced by the AFM image in Figure 1d. The prepared uniform ultrathin microplate may be conducive to the formation of a continuous electrode film. The XRD peaks assigned to the (020), (040) and (060) crystal planes of BP [28,41] are clearly visible, demonstrating the good crystallinity of the microplates, and the corresponding broadening may be related to the trace residues of quaternary ammonium salt in the BP interlayer [29] (Figure 1e). The phosphorus chemical state in the BP microplates has been investigated by XPS. The high intensity of P 2p_1/2_ and P 2p_3/2_ signals ascribed to elemental phosphorus in Figure 1f indicates that the BP microplates are of high quality and hardly oxidized [25,42]. The above results generally illustrate that the high-quality thin-layer BP microplates were produced by the electrochemical intercalation technology, which lays the foundation for the preparation of flexible SCs.

**Figure 2:**
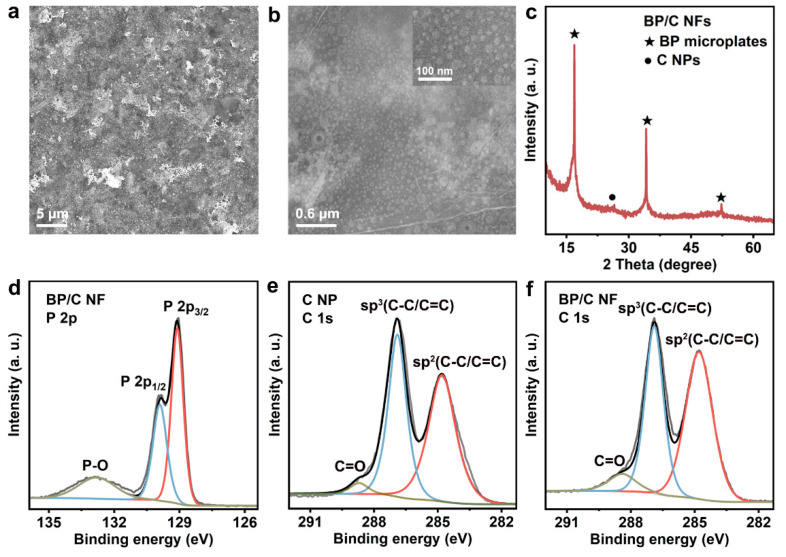
Characterization of BP/C NFs. (**a**,**b**) SEM images of BP/C NFs under different magnifications. (**c**) XRD pattern of BP/C NFs. (**d**–**f**) XPS spectra of (**d**) P 2p for BP/C NF, (**e**) C 1s for C NP, and (**f**) C 1s for BP/C NF.

The electrode of BP/C NFs was prepared by spraying BP microplates and C NPs in ethanol onto the heated ITO-PET film with a high-speed air jet. As seen in the SEM image of Figure 2a, the electrode surface is flat without obvious undulations or cracks. Homogeneously mixed BP microplates and C NPs can be observed in the SEM image at high magnification, and the surface of the BP microplate shows uniformly dispersed small-sized C NPs (Figure 2b). In the XRD pattern of BP/C NFs (Figure 2c), three characteristic peaks from BP and a diffraction peak near 25° from C NPs (Figure S1b) are clearly presented. These hardly shifted characteristic peaks indicate that the BP microplates and C NPs may be dominated by physical mixing [43]. The P2p XPS spectrum of BP/C NF shown in Figure 2d is almost the same as that of the BP microplate (Figure 1f). The slightly elevated P−O peak around 133.0 eV may be due to the slight oxidation of BP/C NF caused by the electrode preparation in air [29,38,44]. The C1s peaks of C NP remain almost unchanged after complexation with BP microplates, as seen from Figure 2e,f. Therefore, there is no obvious electronic state change in the BP/C NFs, and mainly a weak van der Waals force between the BP microplates and C NPs [25,45]. The Raman result (Figure S2) also confirms this inference. In the BP/C NFs, small-sized C NPs are distributed in the layers of the thin BP microplates, forming a certain nano-gap on the substrate to avoid the multiple overlapping of BP plates.

### 3.2. Performance Evaluation of BP/C SC

**Figure 3:**
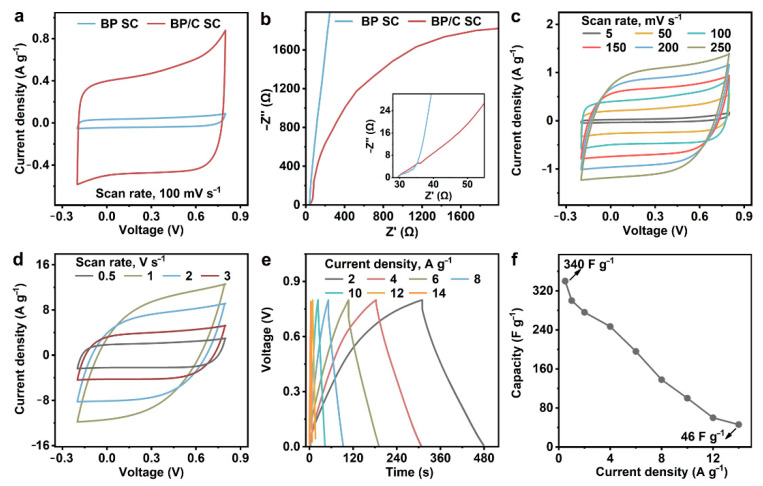
Electrochemical properties of BP/C SC. (**a**) Cyclic voltammetry curves of BP/C SC and BP SC at 100 mV s^−1^. (**b**) Electrochemical impedance spectra of BP/C SC and BP SC with an insert of mid–low-frequency region spectra. (**c**,**d**) Cyclic voltammetry curves of BP/C SC at (**c**) 5–250 mV s^−1^ and (**d**) 0.5–3 V s^−1^. (**e**) Galvanostatic charge–discharge curves of BP/C SC under different current densities. (**f**) Capacity of BP/C SC at 0.5–14 A g^−1^.

The symmetrical flexible supercapacitors BP/C SC and BP SC were fabricated using BP/C NF and BP electrodes on flexible substrates with solid electrolytes, respectively. The electrochemical properties of BP/C SC and BP SC have been evaluated. Cyclic voltammetry (CV) tests were performed within the potential window of −0.2–0.8 V at a scan rate of 100 mV s^−^^1^, and the CV curves of the two supercapacitors are relatively symmetrical rectangles (Figure 3a). The area enclosed by the CV curve of BP/C SC is much larger than that of BP SC, indicating the high mass specific capacitance of BP/C SC. From the two electrochemical impedance spectra in Figure 3b, we see that the difference between BP/C SC and BP SC is mainly concentrated in the mid–low-frequency region. This means that the addition of C NPs to the BP microplates mainly improves the ion diffusivity and has little effect on the electron transfer resistance [12,25]. The CV tests of BP/C SC at different scanning speeds have been studied, and the obtained curves are shown in Figure 3c,d. When the scanning rate is 5 mV s^−1^, the mass specific capacitance of BP/C SC is calculated using Equation (S1) to be 372 F g^−1^, larger than that of BP SC (32.6 F g^−1^). Even at 250 mV s^−1^, the capacity of 171 F g^−1^ and the relative retention of 45.9% are acquired, implying a reasonable electric double-layer capacitance behavior and charge diffusion capacity of BP/C SC. Moreover, the BP/C SC exhibits a similar quasi-rectangular CV curve at high scan rates of 0.5–3 V s^−1^, indicating its good rate capability.

Galvanostatic charge–discharge tests were further performed at different current densities from 2 A g^−1^ to 14 A g^−1^. The corresponding curves are all close to an isosceles triangle, and the ohmic voltage drop in the curves is small (Figure 3e). Thus, the BP/C SC possesses satisfactory capacitance and electrochemical reversibility. The discharge duration is 340 s at 2 A g^−1^, and the discharge voltage is linearly related to the discharge time, which suggests that the Faraday process does not occur. The capacity of BP/C SC in the galvanostatic charge–discharge process calculated using Equation (S2) is shown in Figure 3f. A certain degree of capacity reduction at a high current density may be related to the decrease in the effective contact between the BP microplates and C NPs. Power density is an important parameter of supercapacitors, and the BP/C SC exhibits a maximum power density of 320 W g^−1^ according to Equation (S3).

**Figure 4:**
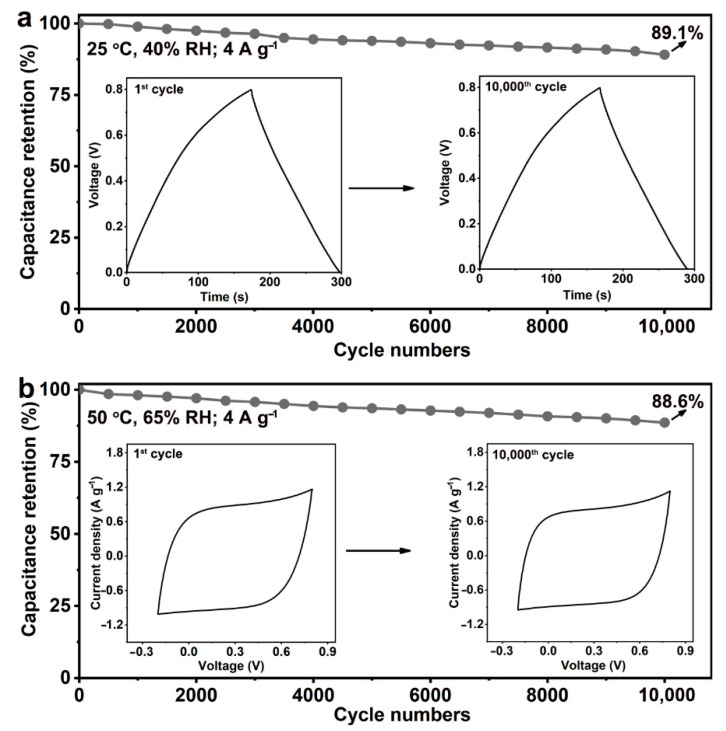
Cycle stability of BP/C SC. (**a**) Stability of BP/C SC at 25 °C and 40% RH during 10,000 cycles; insert, 1st and 10,000th cycle curves. (**b**) Stability of BP/C SC at 50 °C and 65% RH for 10,000 cycles; insert, 1st and 10,000th cycle curves. The current density for both galvanostatic charge–discharge cycles is 4 A g^−1^.

Cycle life is important for supercapacitors; thus, the cycle stability of BP/C SC is investigated under two environmental conditions. As shown in Figure 4a, after 10,000 galvanostatic charge–discharge tests of 4 A g^−1^ at 25 °C and 40% relative humidity (RH), the capacitance retention of BP/C SC is 89.1%, and this may be attributed to the good structural and electrochemical stability. A high relative capacity of 88.6% for BP/C SC is demonstrated in a harsh environment of 65% RH and 50 °C within 10,000 CV tests over 500 h (Figure 4b), which is similar to the results gained in a conventional environment (Figure 4a). Therefore, the assembled BP/C SC exhibits admirable environmental stability and possesses the ability to withstand high-humidity and high-temperature environments.

**Figure 5:**
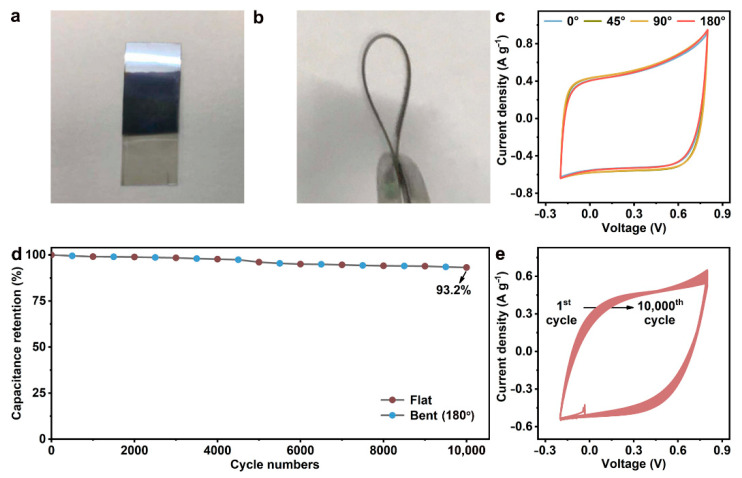
Flexible capacity of BP/C SC. (**a**,**b**) White light photographs of BP/C SC in (**a**) flat and (**b**) bent states. (**c**) Cyclic voltammetry curves at different bending degrees with a scan rate of 150 mV s^−1^. (**d**) Capacitance retention in 10,000 flat-bend tests under 100 mV s^−1^. (**e**) Cyclic voltammetry curves during 10,000 flat-bends at 100 mV s^−1^.

To meet the demands of wearable electronics, supercapacitors are urgently required to maintain a sufficient electrochemical capability after repeated bending [1,23]. The flat BP/C SC (Figure 5a) exhibits intact structures after bending (Figure 5b). Then, the CV tests at bending angles of 0°, 45°, 90°, and 180° are performed, and the obtained curves shown in Figure 5c almost overlap. This proves that the structural bending of the BP/C SC does not affect its electrochemical performance. In 10,000 flat-bend tests with a bending angle of 180°, the BP/C SC achieves a high capacitance retention of 93.2%, and its CV curves remained basically unchanged, as seen from Figure 5d,e. Compared with the reported devices, the BP/C SC possesses considerable supercapacitor performance (Table 1). Thus, the designed BP/C SC with a reasonable BP/carbon composite structure has satisfactory mechanical toughness and good wearable prospects. Such stable long-cycle electrochemical properties promote the BP/C SC as a good alternative for solar energy harvesting. Our proposed method is simple and easy to operate, and enables batch preparation, which has high application potential compared with other manufacturing approaches [13,26,36].

## 4. Conclusions

A feasible strategy of embedding 0D carbon nanospheres into the 2D interlayers of BP ultrathin microplates to form nanoframes is proposed to promote charge and ion diffusion on BP. In the obtained BP/C NF, the BP microplates and C NPs are only a simple physical mixture. Thanks to the micro–nano composite structure formed from the 2D microplates and 0D nanoparticles, the flexible BP/C SC exhibits a capacity of 372 F g^−1^ at 5 mV s^−1^ and ca. 90% capacitance retentions after 10,000 cycles under 4 A g^−1^ charge–discharge test and repeated flat-bend. Our approach to building BP/C nanoframes is not only helpful for the design of supercapacitors, but also offers nanomaterials for solar thin devices, secondary ion batteries electrodes, and separation membranes.

## Figures and Tables

**Table 1:** Performance comparisons of the BP/C SC and the reported BP-based and other supercapacitors.

Electrode Material	Capacitance (F g^−1^) at a Scan Rate (mV s^−1^)	Stability Test	Capacitance Retention	Ref.
BP/C	372 (5)	4 A g^−1^ galvanostatic	89.1% (10,000th)	This work
		Flat-bend	93.2% (10,000th)	
BP nanoflakes	45.8 (10)	Flat-bend	84.5% (1000th)	[37]
BP sponges	80 (10)	0.1 V s^−1^ cycle	80% (15,000th)	[26]
BP/GO	104.4 (250)	5 A g^−1^ galvanostatic	92.7% (5000th)	[46]
BP/G	37.5 (5)	Flat-bend	89.5% (2000th)	[27]
BP/PANI	354 (300)	0.3 A g^−1^ galvanostatic	87% (175th)	[33]
N,P,S-HCS	31.3 (500)	10 A g^−1^ galvanostatic	95% (1000th)	[16]
PGO/CC	211.7 (1000)	10 A g^−1^ galvanostatic	89.3% (10,000th)	[47]
Ni-Mn-S@NiCo_2_S_4_	939 (1000)	5 A g^−1^ galvanostatic	90.3% (5000th)	[20]

## Data Availability

Not applicable.

## Supplementary Materials

The following supporting information can be downloaded at: https://www.mdpi.com/article/10.3390/nano12193311/s1, instruments and calculations of electrochemical properties; Figure S1: characterization of C NPs. (a) SEM image. (b) XRD pattern; Figure S2: Raman scattering spectra of BP microplates, C NPs, and BP/C NFs; supplementary references: S1–S3.
