# Supplementary material for: Black Phosphorus/Carbon Nanoframes for Efficient Flexible All-Solid-State Supercapacitor
Source: Nanomaterials (Basel). 2022 Sep 23;12(19):3311. doi: 10.3390/nano12193311 (PMC9565270; doi:10.3390/nano12193311)
Supplement: Supplementary file 1 [file nanomaterials-12-03311-s001.zip › nanomaterials-1916507-supplementary.pdf]

nanomaterials

Supplementary Materials

Black Phosphorus/Carbon Nanoframes for Efficient Flexible All-Solid-State Supercapacitor

Zunbin Duan [1,†], Danni Liu [1,†], Zhaoer Ye [1,3], Caixia Sun [1,4], Zikun Wang [1,3], Kezhen Chen [1,5], Yang Li [1], Hao Huang [1], Xiaoliang Zeng [2], Jiahong Wang [1,*], Rong Sun [2,*] and Xue-Feng Yu [1,*]

[1] Shenzhen Engineering Center for the Fabrication of Two-Dimensional Atomic Crystals, Shenzhen Institute of Advanced Technology, Chinese Academy of Sciences, Shenzhen 518055, China
[2] Shenzhen Institute of Advanced Electronic Materials, Shenzhen Institute of Advanced Technology, Chinese Academy of Sciences, Shenzhen, 518055, China
[3] Nano Science and Technology Institute, University of Science and Technology of China, Suzhou 215125, China
[4] Department of Hematology, Zhanjiang Central Hospital, Guangdong Medical University, Zhanjiang 524045, China
[5] University of Chinese Academy of Sciences, Beijing 100049, China
* Correspondence: jh.wang1@siat.ac.cn (J.W.); rong.sun@siat.ac.cn (R.S.); xf.yu@siat.ac.cn (X.-F.Y.)
† These authors contributed equally to this work.

1. Instruments and calculations of electrochemical properties

Scanning electron microscopy (SEM) was performed on a Zeiss Supra 55 high-resolution field-emission scanning electron microscope at 2.0 kV and a distance of 5.0 mm. Transmission electron microscopy (TEM) was acquired on a Tecnai G2 F20 S-Twin transmission electron microscope at 200 kV. Atomic force microscopy (AFM) was performed on an Oxford AR Cypher S atomic force microscope using the tapping mode. Optical microscopy was employed to observe the dispersed BP microplates. Powder X-ray diffraction (XRD) was performed on a Rigaku SmartLab diffractometer at 40 kV and 30 mA. X-ray photoelectron spectroscopy (XPS) was carried out on a Thermo Fisher ESCALab 250Xi spectrometer with Al Kα radiation. The BP microplates were prepared by using an IT6123B workstation. Raman scattering microscopy was conducted on the Horiba Jobin-Yvon Lab Ram HR VIS Raman microscope (633 nm laser as the excitation; RT). The spraying process was performed using an HF-600 air pump sprayer with an airbrush. The electrochemical performance of the supercapacitors BP/C SC and BP SC was evaluated on a CHI-760E workstation (China, Shanghai). The cyclic voltamograms (CV) and galvanostatic charging-discharging curves were obtained to investigate the electrochemical properties. The electrochemical evaluations were performed in two environmental conditions, including a conventional environment of 25 °C and 40% RH and a harsh environment with a high temperature of 50 °C and a high relative humidity of 65%. The cycle stability of BP/C SC was evaluated by using a 10,000 galvanostatic charge-discharge cycles under a high current density of 4 A g^{-1}. The bending angle is 180° in 10,000 flat-bend cycles.

The specific capacitance of the device was calculated from the CV curves with the following Eq. S1,

$$C_m = \int i(V)dV/mv\Delta V \quad \text{(S1)}$$

where C_m is the mass capacitance of the device, m is the mass of the electrode material, v is the scanning rate, and ΔV is the potential window.

The specific capacitance of BP/C SC in galvanostatic charging-discharging process was calculated by using Eq. S2,

$$C_m = \frac{I\Delta t}{m\Delta V} \quad \text{(S2)}$$

where I is the current density, and Δt is the discharge time.

The power density of BP/C SC is calculated by using the following Eq. S3,

$$P = I\Delta V/m \quad \text{(S3)}$$

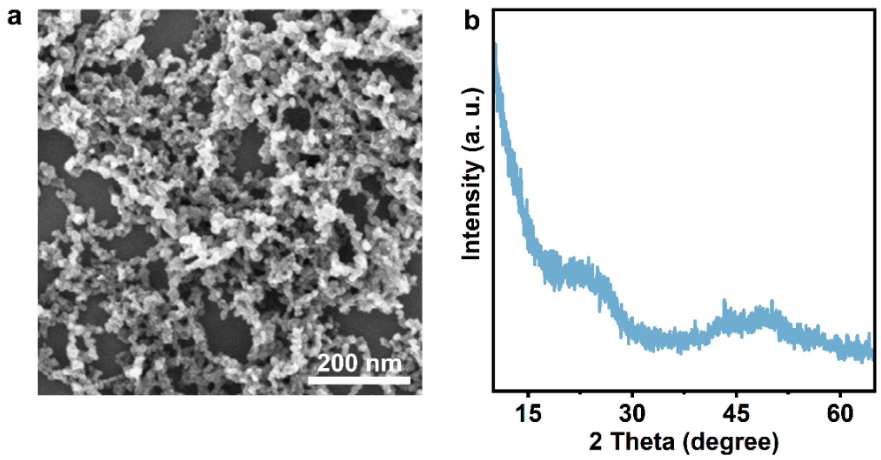

Figure S1. Characterization of C NPs. (a) SEM image. (b) XRD pattern.

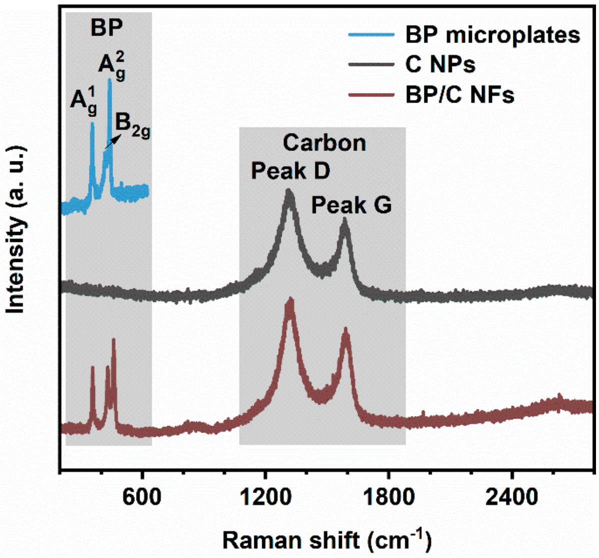

Figure S2. Raman scattering spectra of BP microplates, C NPs, and BP/C NFs.

In the Raman spectra shown in Figure S2, the BP/C NFs possess characteristic peaks of BP near 360.6 cm^{-1} of A_g^1, 439.7 cm^{-1} of B_{2g}, and 466.2 cm^{-1} of A_g^1 [S1, S2]and characteristic peaks of carbon around 1365 cm^{-1} of peak D and 1596 cm^{-1} of peak G [S3], which are not much different from BP microplates and C NPs alone. This may mean that the BP microplates and C NPs are only physically mixed in the BP/C NFs.

Supplementary references

S1. Zeng, L.; Zhang, X.; Liu, Y.; Yang, X.; Wang, J.; Liu, Q.; Luo, Q.; Jing, C.; Yu, X.; Qu, G.; Chu, P. K.; Jiang, G. Surface and interface control of black phosphorus. *Chem* **2022**, *8*, 632-662.

S2. Li, L.; Yu, Y.; Ye, G. J.; Ge, Q.; Ou, X.; Wu, H.; Feng, D.; Chen, X. H.; Zhang, Y. Black phosphorus field-effect transistors. *Nat. Nanotechnol.* **2014**, *9*, 372-377.

S3. Duan, Z.; Wang, Y.; Bian, S.; Liu, D.; Zhang, Y.; Zhang, X.; He, R.; Wang, J.; Qu, G.; Chu, P. K. Size-dependent flame retardancy of black phosphorus nanosheets. *Nanoscale* **2022**, *14*, 2599-2604.
